# Distinct summer and winter bacterial communities in the active layer of Svalbard permafrost revealed by DNA- and RNA-based analyses

**DOI:** 10.3389/fmicb.2015.00399

**Published:** 2015-04-30

**Authors:** Morten Schostag, Marek Stibal, Carsten S. Jacobsen, Jacob Bælum, Neslihan Taş, Bo Elberling, Janet K. Jansson, Philipp Semenchuk, Anders Priemé

**Affiliations:** ^1^Department of Geosciences and Natural Resource Management, Center for Permafrost, University of CopenhagenCopenhagen, Denmark; ^2^Geological Survey of Denmark and Greenland (GEUS)Copenhagen, Denmark; ^3^Department of Biology, University of CopenhagenCopenhagen, Denmark; ^4^Department of Environmental Sciences, Aarhus UniversityDenmark; ^5^Center for Biological Sequence Analysis, Technical University of DenmarkLyngby, Denmark; ^6^Ecology Department, Lawrence Berkeley National LaboratoryBerkeley, CA, USA; ^7^Biological Sciences Division, Pacific Northwest National LaboratoryRichland, WA, USA; ^8^Department of Arctic and Marine Biology, University of TromsøTromsø, Norway

**Keywords:** permafrost active layer, seasonal variation, bacterial community structure, 16S rRNA gene, Arctic

## Abstract

The active layer of soil overlaying permafrost in the Arctic is subjected to dramatic annual changes in temperature and soil chemistry, which likely affect bacterial activity and community structure. We studied seasonal variations in the bacterial community of active layer soil from Svalbard (78°N) by co-extracting DNA and RNA from 12 soil cores collected monthly over a year. PCR amplicons of 16S rRNA genes (DNA) and reverse transcribed transcripts (cDNA) were quantified and sequenced to test for the effect of low winter temperature and seasonal variation in concentration of easily degradable organic matter on the bacterial communities. The copy number of 16S rRNA genes and transcripts revealed no distinct seasonal changes indicating potential bacterial activity during winter despite soil temperatures well below −10°C. Multivariate statistical analysis of the bacterial diversity data (DNA and cDNA libraries) revealed a season-based clustering of the samples, and, e.g., the relative abundance of potentially active *Cyanobacteria* peaked in June and *Alphaproteobacteria* increased over the summer and then declined from October to November. The structure of the bulk (DNA-based) community was significantly correlated with pH and dissolved organic carbon, while the potentially active (RNA-based) community structure was not significantly correlated with any of the measured soil parameters. A large fraction of the 16S rRNA transcripts was assigned to nitrogen-fixing bacteria (up to 24% in June) and phototrophic organisms (up to 48% in June) illustrating the potential importance of nitrogen fixation in otherwise nitrogen poor Arctic ecosystems and of phototrophic bacterial activity on the soil surface.

## Introduction

Permafrost covers approximately 25% of the land area in the northern hemisphere not covered by ice sheets (Zhang et al., 2008), and the surface of an even larger fraction of the land area undergoes seasonal freezing. The Arctic is greatly affected by global warming (IPCC, 2013) and an increase in air temperature is expected to increase soil temperature, increase plant-growing season, enhance permafrost thaw and hence increase the depth of the active layer overlaying permafrost. Permafrost is estimated to contain 1024 Pg of organic carbon in the uppermost 3 m corresponding to ~30% of the global soil organic carbon pool (Tarnocai et al., 2009). When permafrost thaws, it is transformed into an active layer of soil where the microbial activity is likely to increase (Elberling et al., 2013; Knoblauch et al., 2013). Thus, microbial degradation of organic matter in recently formed as well as old active layer soils may increase when soil temperature increases, leading to elevated release of CO_2_ to the atmosphere (Schuur et al., 2009) and potentially profound changes to the global climate (DeConto et al., 2012).

Even though the Arctic is currently greening due to expansion of, e.g., scrub (Elmendorf et al., 2012), plant cover on the tundra often allows enough photosynthetically active radiation to reach the soil surface for surface-dwelling *Cyanobacteria* and other phototrophic microorganisms to proliferate. Thus, input of organic matter produced by phototrophic microorganisms may be of ecological importance in Arctic soils. Also, Arctic plants and soil microorganisms are generally assumed be limited by availability of nitrogen (Elser et al., 2007). Arctic soils receive low rates of atmospheric nitrogen deposition (Dentener et al., 2006), which makes fixation of atmospheric nitrogen by free-living and plant-associated microorganisms the primary input of nitrogen in terrestrial Arctic ecosystems (Reed et al., 2011).

At lower latitudes, seasonal fluctuations in soil moisture, temperature and nutrients can result in distinct changes in bacterial numbers and community structure (e.g., Bardgett et al., 1999; Cruz-Martinez et al., 2009; Rasche et al., 2010). In contrast to surface soils at lower latitudes and permafrost soil, active layer soils in the Arctic are subjected to temperatures below −10°C for several months during winter, which may affect the activity of soil microorganisms (Ayala-del-Río et al., 2010; Mykytczuk et al., 2013). The subzero winter temperatures are replaced by temperatures above 0°C during the short summer leading to pronounced seasonal variation in temperature of active layer soil. In addition, active layer microorganisms may experience seasonal variation in, e.g., unfrozen water content, osmolarity of liquid water, exudates from plant roots, photosynthetically active radiation, and UV radiation.

Despite this strong seasonality, only few studies have investigated seasonal changes in the active microbial communities in Arctic soils (e.g., Lipson and Schmidt, 2004; McMahon et al., 2011; Kuffner et al., 2012; Lazzaro et al., 2012), and none of these involved more than four time points. This leaves a gap in our understanding of changes in the microbial community and their activity in this environment over the course of a whole year.

Soil bacterial communities may be investigated using DNA sequencing technologies involving sequencing of metagenomes or PCR amplified 16S rRNA gene fragments. The latter has been widely used to study bacterial community structure in a range of soils (e.g., Roesch et al., 2007; Lauber et al., 2009) and allows for more sequencing depth of the 16S rRNA gene compared to metagenome sequencing. Also, Hultman et al. (2015) found very similar bacterial phylogenetic composition of Alaskan active layer soil when comparing sequencing of 16S rRNA gene amplicons and metagenomes.

Bacterial communities in Arctic soils have a structure similar to those found in soil environments elsewhere (Neufeld and Mohn, 2005; Chu et al., 2010). Since studies of the 16S rRNA gene only reveals the bulk diversity of a bacterial community and do not distinguish between active and inactive bacteria, 16S rRNA transcripts have been used as a marker of potentially active bacteria (Baldrian et al., 2012; Kotik et al., 2013; Tveit et al., 2013; Stibal et al., 2015; and see review by Blazewicz et al., 2013 discussing the interpretation of 16S rRNA transcripts). Metatranscriptomic analysis of the upper layer of a Svalbard peatland revealed that rRNA from bacteria comprised 70–80% of all rRNA from microorganisms, i.e., bacteria, archaea and eukaryotes (Tveit et al., 2013). This finding illustrates the importance of bacteria in the turnover of organic matter and nutrient cycling in Arctic active layer soils.

We hypothesized that (i) extremely low soil temperature during winter will have pronounced impact on the abundance and structure of the bacterial community, (ii) this impact will be more pronounced on the potentially active part of the bacterial community with hardly any potentially active bacteria detectable during the peak of winter, (iii) seasonal changes in the availability of easily degradable organic carbon and nitrogen will influence the abundance and structure of the potentially active part of the bacterial community, (iv) freezing/thawing will influence the bacterial community structure in autumn and spring, (v) nitrogen-fixing bacteria are common in this presumed nitrogen-limited environment and show profound seasonal changes due to changes in temperature and in-coming photosynthetically active radiation, and (vi) phototrophic microorganisms are common during summer. Twelve soil cores collected at monthly intervals in Adventdalen, Svalbard, were chosen to represent seasonal variations in active layer permafrost. To our knowledge, this is the first study to investigate the full annual cycle of bacterial diversity in Arctic soil using co-analysis of the 16S rRNA gene and 16S rRNA transcripts.

## Materials and methods

### Site description and soil sampling

Soil samples were collected in an Arctic tundra in Adventdalen, Svalbard, (78.172°N, 16.062°E), dominated by *Salix polaris* and *Luzula arcuata* subsp. *confusa* (Cooper et al., 2011). At Svalbard Airport, ca. 16 km from the sampling site, the mean annual temperature for 2010–2011 was −3.7°C (data retrieved from Norwegian Meteorological Institute, Svalbard Lufthavn^1^). The cables to the temperature sensor at the sampling site were destroyed before the termination of the sampling period, so soil temperature at 25 cm soil depth was obtained 4 km from the site (see http://geo.ngu.no/kart/permafrost_svalbard/). During the short interval of their concurrent functioning, temperature values from the sensors at similar soil depth at the two sites were almost identical (data not shown). Snow covered the ground from 11th of October 2010 to 14th of May 2011 (Norwegian Meteorological Institute, Svalbard Lufthavn). Within a sampling area of 3 × 3 m, a total of 12 soil cores were collected at approximately monthly intervals from 17th of June 2010 to 31st of May 2011 (Table 1). Thus, each of our sampling dates is represented by only a single soil core, which reflects the difficulties involved in drilling soil cores during a full year in the Arctic. All cores were collected using a motorized hand drill with an expandable drill string and a 50-cm drill head with a diameter of 4.8 cm. Each core was pushed out of the drill head directly into a sterile plastic bag and kept cold with cooling elements in a thermo box and frozen to −18°C within 8 h of collection. Prior to analysis, the soil cores were stored for up to 8 months at −18°C, which we believe is an appropriate storage temperature since Sessitsch et al. (2002) found no significant difference in the rRNA content between soil samples stored at −20°C and fresh samples over a period of 4 weeks.

**Table 1:** **Sampling dates and soil physical-chemical parameters**.

**Sampling date**	**Depth of soil core (cm)**	**Soil temperature (°C)**	**Soil water content (% of dwt)**	**pH**	**NO^-^_3_ (mg l^−1^)**	**NH^+^_4_(mg l^−1^)**	**DOC (mg l^−1^)**	**DON (mg l^−1^)**	**Total C (%)**	**Total N (%)**
17/6-2010	0–24	4.6	30	5.0	0.23	2.1	26	3.0	2.9	0.3
22/7-2010	1–11	5.2	25	5.2	0.33	1.6	36	2.2	2.4	0.2
13/8-2010	0–18	4.9	33	4.9	0.33	1.8	66	4.1	4.1	0.3
16/9-2010	0–7	0.2	31	4.7	0.30	1.9	38	2.5	2.9	0.2
12/10-2010	0–10	0.5	81	5.6	0.34	2.6	87	8.3	7.5	0.5
13/11-2010	0–6	−6.5	62	5.1	0.30	2.0	90	7.0	5.8	0.4
2/12-2010	0–8	−7.2	91	5.6	0.43	4.4	85	10.7	8.7	0.6
17/1-2011	0–10	−13.5	68	4.6	0.35	2.7	146	8.1	6.3	0.5
19/2-2011	0–10	−14.3	35	5.3	0.82	3.4	66	5.0	2.7	0.2
11/3-2011	0–10	−7.9	39	5.4	0.38	1.7	60	4.3	3.4	0.2
3/5-2011	0–10	−3.0	94	5.3	0.55	3.2	117	8.0	5.0	0.3
31/5-2011	0–18	−0.2	31	5.0	0.31	1.1	40	2.8	2.6	0.2

*Soil temperature was measured at 25 cm depth. DOC, dissolved organic carbon; DON, dissolved organic nitrogen*.

### Soil characterization

Upon arrival to Copenhagen, the frozen soil cores were divided into 5-cm pieces by a sterile chisel, of which about 50 grams was used for this study. A subsample from each soil core was thawed at 4°C. Twenty gram (wet weight) of the thawed soil was mixed with 20 ml milliQ water and shaken for 12 h at room temperature before measurement of pH with a PHM 80 pH meter (Radiometer, Copenhagen, Denmark). Water was drained from the thawed core at 0°C and acidified prior to analysis of dissolved organic carbon using 2 M HCl to remove inorganic carbon. Dissolved organic carbon was analyzed using a Shimadzu TOC 5000A analyzer (Shimadzu Scientific Instruments, Kyoto, Japan). For determination of the total dissolved organic nitrogen concentration, 4 ml of the soil solution were mixed with 4 ml 3% w/v sodium peroxosulfate in 0.15M NaOH in a 12-ml Kimax tube. The solution was then incubated at 100°C for 60 min, cooled to room temperature and analyzed on FiaStar 5000 (Foss, Höganäs, Sweden). The nitrate and ammonium concentrations in the soil solution were also measured on FiaStar 5000, without the boiling step, following the manufacturer's instructions. Soil water content was estimated by heating 15 g of wet soil at 120°C for a minimum of 12 h, until the weight of the dried soil sample was stable. Finally, total nitrogen and total carbon contents were determined in 0.3 g of soil sample by combusting the sample for 2 min at 850°C in a Leco TruSpec Carbon Nitrogen Determinator Model 630-100-100 (Leco, St Joseph, MI, USA).

### Coextraction of DNA and RNA

Prior to DNA/RNA extraction, 5 g of soil was aseptically removed from each sample by hammer and chisel, and freeze dried to remove any soil water. After freeze-drying, samples were homogenized, and was randomly sampled for DNA and RNA extraction. To avoid any potential effect of RNase activity all glassware were furnaced at 240°C for 4 h prior to use, and only certified DNase free plasticware were used. All solutions were prepared with diethylpyrocarbonate (DEPC)-treated water. During the extraction procedure, the samples were kept on ice unless stated otherwise. Three replicate subsamples of the freeze-dried samples were used for the co-extraction of DNA and RNA, resulting in a total of 36 samples. To lyse the cells, 0.5 g of soil was transferred to a bead-containing tube (1.4 mm Ceramic Bead Tube, MO BIO Laboratories, Carlsbad, CA, USA) and extraction was performed as described by Nicolaisen et al. (2008) bead beating twice for 20 s at speed 5 in a FastPrep bead beater (FastPrep FP120, MP Biomedicals, CA, USA) with a cooling step on ice in between. The resulting nucleic acid solution was cleaned using NucleoSpin RNA Clean-Up Kit (Macherey-Nagel, Düren, Germany) following the manufacturer's instructions. The final volume of extract was 20 μl, of which 10 μl was used for DNA and 8 μl for RNA analysis. DNA samples were stored at −80°C until further analysis. RNA samples were immediately DNase treated using the RTS DNase Kit (MO BIO Laboratories, Carlsbad, CA, USA) following the manufacturer's instructions. After DNA removal, 6 μl of pure RNA template were reverse transcribed using random hexamer primers (Thermo Scientific, Schwerte, Germany) and RevertAid Reverse Transcriptase (Thermo Scientific). Reverse transcription was performed by incubation at 25°C for 10 min, 50°C for 30 min and 85°C for 5 min. cDNA samples were stored on −80°C until further analysis.

### PCR and sequencing of 16S rRNA gene DNA and cDNA

The V4 region of the 16S rRNA gene in all DNA and cDNA samples was amplified using region specific primers [515F: GTGCCAGCMGCCGCGGTAA, and 806R: GGACTACHVGGGTWTCTAAT (Caporaso et al., 2011)] suitable for Illumina HiSeq sequencing. These primers have a high coverage of different bacterial and archaeal phyla compared to other frequently used primers targeting the 16S rRNA gene (Mori et al., 2014) and covers the V4 region of the 16S rRNA gene, which is particularly suited for short read [120–130 bp] 16S rRNA gene sequencing studies (Soergel et al., 2012; Ghyselinck et al., 2013) The primers used were modified from Caporaso et al. (2012), since barcodes of 8 bp instead of 12 bp were used. The PCR reactions contained 0.25 μl TaKaRa Ex Taq proofreading DNA polymerase (TaKaRa Bio, Shiga, Japan), 5 μl buffer, 4 μl dNTP mix (0.8 μM final concentration), 2 μl of each forward and reverse primer (0.4 μM final concentration), 35.75 μl DEPC treated dH_2_O and 1 μl of template DNA/cDNA. The PCR protocol was: 94°C for 3 min to denature the DNA/cDNA and activate the polymerase, 25 cycles of 94°C for 45 s, 50°C for 60 s, and 72°C for 90 s, and finally 10 min at 72°C to ensure complete extension of the amplicons. The PCR products were purified using carboxyl-coated magnetic beads (SPRI beads, Agencourt AMPure XP, Agencourt, Beverly, MA, USA) following the manufacturer's description. The concentration of the PCR products was measured using a dsDNA HS Assay Kit and the Qubit fluorometer (Life Technologies, Carlsbad, CA, USA). All PCR products were also run on a 1.5% agarose gel stained with ethidium bromide to check if PCR artifacts were removed. No PCR products were detected from DNase treated RNA samples. Prior to sequencing, 50 ng PCR products from each sample were pooled into a DNA and a cDNA sample. The pooled samples were then sent to Argonne National Laboratory (Lemont, IL, USA) for Illumina HiSeq 2000 single-end 150 bp sequencing.

### PCR quantification of 16S rRNA gene and transcript copies

Quantitative PCR was performed to estimate the numbers of 16S rRNA gene and transcript copies in the soil samples. Three replicate PCR reactions were performed on each DNA/cDNA sample using the primers 341F (5′-CCTACGGGAGGCAGCAG-3′) and 518R (5′-ATTACCGCGGCTGG-3′) (Lane, 1991). For producing a standard, *E. coli* K27 was grown in Luria-Bertani medium for 2 days at room temperature to a desired density and DNA was extracted. A dilution series from 8.45 × 10^3^ to 8.45 × 10^6^ 16S rRNA gene copies per μl was used as standards. PCR reactions were performed using SyGreen Mix Lo-ROX (PcrBioSystems, London, UK) and were carried out on a CFX96 Touch qPCR system (Bio-Rad, Hercules, CA, USA). The PCR setup involved 10 μl SyGreen Mix, 0.8 μl of each primer (final concentration 0.4 μM), 1 μl template DNA/cDNA, and 7.4 μl of DEPC treated dH_2_O to a final volume of 20 μl. PCR conditions were: 95°C for 30 s and 40 cycles of 30 s at 95°C, 30 s at 50°C, 30 s at 72°C, and 10 s at 82°C. The reaction was completed with 72°C for 6 min for final elongation and followed by a melting curve analysis going from 55°C to 98°C in 0.5°C increments.

### Analyses of DNA and cDNA sequences

Illumina-based 16S rRNA gene sequencing may be analyzed using a choice of different bioinformatic pipelines like UPARSE (Edgar, 2013) or mothur (Schloss et al., 2009), but we chosed the widely used and well-referenced QIIME Pipeline (Caporaso et al., 2010). Low quality sequences were removed on the basis of the following criteria: (1) sequences with more than three consecutive low quality base calls; (2) sequences that contained less than 75% high quality base calls of total sequence; (3) sequences with more than one mismatch within the barcode; (4) sequences with a phred score below 3 (Bokulich et al., 2012).

Because the cDNA sequences on average were longer than the DNA sequences (151 bases vs. 123 bases), cDNA sequences were shortened *in silico* to achieve the same average length of cDNA and DNA derived sequences. In total, 5,154,024 sequences were obtained from the DNA samples after quality filtering, ranging from 84,919 to 296,684 per sample (average 143,167 sequences per sample). From the cDNA samples, 32,885,340 sequences were obtained after quality filtering, ranging from 309,764 to 1,152,022,274 sequences per sample (average 913,481 per sample). A total of 8326 and 11,105 different OTUs were detected from the DNA and cDNA samples, respectively, after 97%-matching to the Greengenes database and removal of singletons.

Sequences of sufficient quality were aligned to the Greengenes database (DeSantis et al., 2006) and operational taxonomic units (OTUs) were defined by a 97% or larger identity to database sequences. Non-matching sequences were discarded, corresponding to 17 and 26% of the sequences for DNA and cDNA samples, respectively. The Greengenes database was also used to assign taxonomic identity to each OTU. OTU tables were rarefied 1000 times to an even depth of 84,919 sequences per samples and merged into a single OTU table. Final OTU tables were trimmed by removing all singletons so only OTUs represented by more than one sequence were used for further analysis. Furthermore, chimeras were removed using USEARCH 6.1 (Edgar et al., 2011) with default settings. The sequences have been deposited at the European Nucleotide Archive with the study accession number PRJEB7947.

### Statistical analysis

Multivariate statistical analysis was employed to explain variations in the data and to test for significant effects of environmental parameters on the bacterial community structure. All analyses were performed using the multivariate data analysis software Canoco 5 (ter Braak and Šmilauer, 2012).

The relative abundance of each phyla or OTU was log transformed and used as response (explained) data in the analysis, while sampling date (as day of year), soil depth, sample storage time, soil temperature, water content, pH, ratio of total carbon to total nitrogen, dissolved organic carbon, total dissolved nitrogen, nitrate and ammonium were used as quantitative environmental (explanatory) variables. All the environmental data except pH, storage time, soil depth, and day of the year were log transformed prior to analysis (Ramette, 2007).

The data were analyzed with a combination of constrained and unconstrained ordination methods in order to account for both total variation in the data and variation explainable by the environmental data. Detrended correspondence analysis and detrended canonical correspondence analysis were used to determine the length of the gradient along the first ordination axis in order to select the appropriate method for unconstrained and constrained ordination of the data, respectively.

Principal component analysis was employed to analyze the total variation in the DNA or cDNA sequence data. Redundancy analysis was used to calculate the statistical significance of multiple environmental variables as controls of the bacterial community structure. The following redundancy analysis setup was used in the analysis: 9999 Monte Carlo permutations in the restricted mode for time series and manual forward selection. The *P*-values were corrected for type 1 error using false discovery rate.

## Results

### Soil characteristics

The soil temperature ranged from −14.3°C to 5.2°C during the sampling period, while pH ranged between 4.6 and 5.6 (Table 1). The concentration of dissolved organic carbon ranged from 26 mg l^−1^ in June 2010 to 146 mg l^−1^ in January 2011 and was higher during winter. A similar pattern was observed for dissolved organic nitrogen. Additional soil physical and chemical parameters are shown in Table 1.

### 16s rRNA gene and transcript copy number

**Figure 1:**
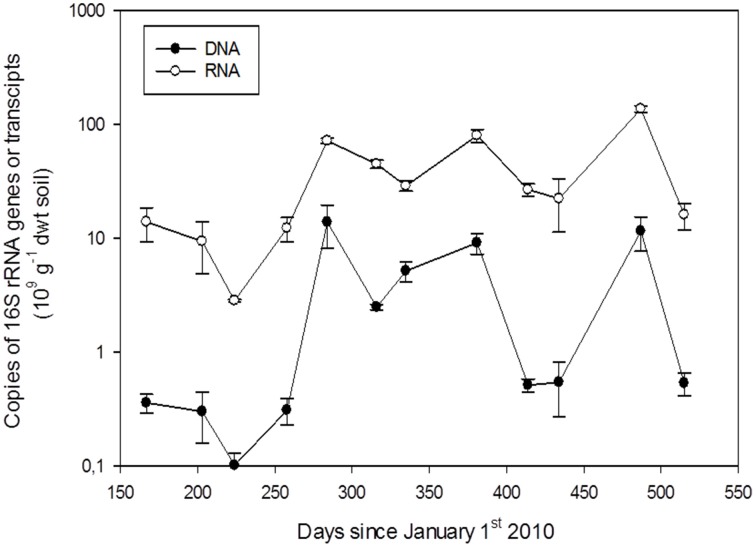
**The seasonal variation in copy numbers of 16S rRNA transcripts and genes during the study period**. Error bars denote standard error of the mean (*n* = 3 qPCR performed on replicate extractions of nucleic acids from the same soil core).

Quantitative PCR was employed to investigate seasonal changes in numbers of bacteria (DNA) and potentially active bacteria (RNA). 16S rRNA gene copy numbers ranged between 0.1 ×10^9^ and 12 ×10^9^ DNA copies g^−1^ dwt soil, while 16S rRNA transcript copy numbers ranged between 2.8 ×10^9^ and 136 ×10^9^ RNA copies g^−1^ dwt soil without showing any distinct seasonal variation (Figure 1). The average ratio of 16S rRNA transcript to gene copy numbers for the study period was 33 ± 8.2 (*n* = 12) (Table 2), with no significant difference (*P* > 0.05) between samples obtained during winter (defined as sampling days with subzero soil temperature) and summer. The ratio varied widely between bacterial groups from 1.1 ± 0.51 for *Gemmatimonadetes* to 141 ± 32 for *Deltaproteobacteria* (Table 2).

**Table 2:** **Ratio of copy numbers of 16S rRNA transcripts to 16S rRNA genes assigned to the numerically dominant bacterial phyla and proteobacterial classes**.

**Phylum or class**	**Ratio RNA:DNA copy numbers (average ± standard error of the mean)**
*Deltaproteobacteria*	141 ± 32^**^
*Firmicutes*	98 ± 33^*^
*Cyanobacteria*	93 ± 26^*^
*Planctomycetes*	71 ± 29
*Alphaproteobacteria*	65 ± 15
*Bacteroidetes*	53 ± 14
*Chloroflexi*	44 ± 15
*Betaproteobacteria*	35 ± 10
Average	33 ± 8.2
*Gammaproteobacteria*	32 ± 9.3
Other phyla (average)	30 ± 5.4
*Acidobacteria*	28 ± 7.5
*Actinobacteria*	26 ± 8.4
*AD3*	20 ± 6.9
*Verrucomicrobia*	16 ± 4.3
*Gemmatimonadetes*	1.1 ± 0.51^**^

“Other phyla” is the sum of numerically uncommon and rare phyla. Values are average ± standard error of the mean for the 12-months sampling period (n = 12).

* Indicates that the ratio is significantly different from the average ratio (P < 0.05);

** *Indicates that the ratio is significantly different from the average ratio (P < 0.01)*.

### Bacterial diversity

**Figure 2:**
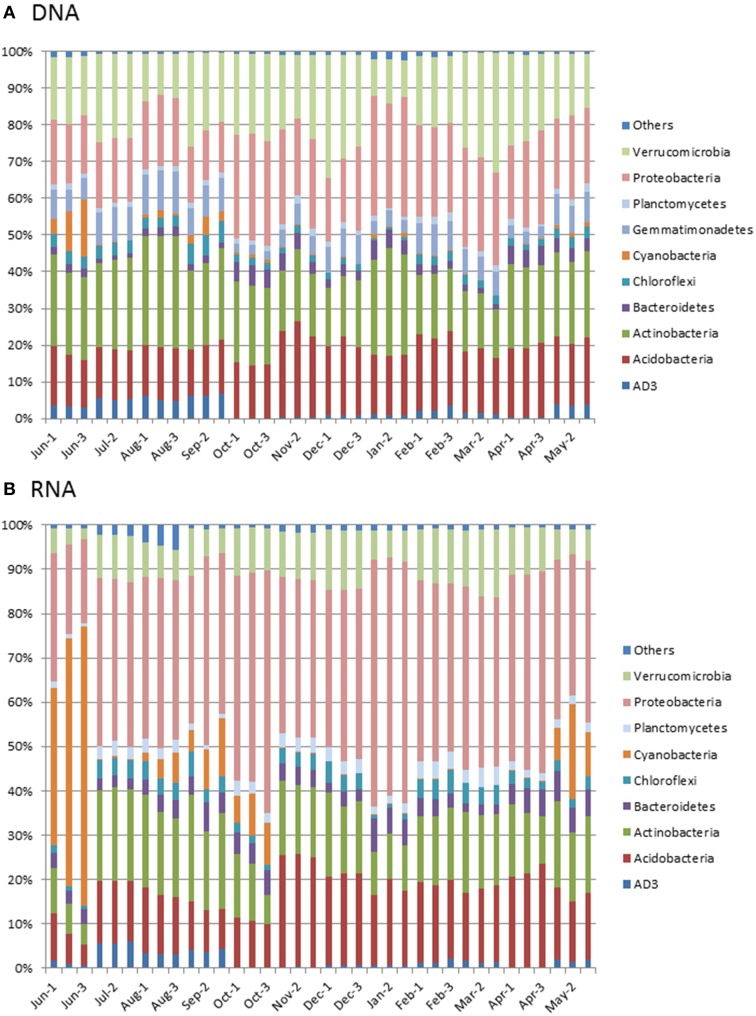
**Relative abundance of bacterial and archaeal phyla based on sequencing of 16S rRNA gene fragments at DNA level (A) and RNA level (B)**. “Others” represent phyla with relative abundance of <1%. Replicates represent three extractions of nucleic acids from the same soil core.

Sequencing of 16S rRNA gene fragments and transcripts revealed the diversity and structure of the bacterial communities. We found no differences in alpha-diversity measures (richness, Chao1, Shannon-Weaver index, and evenness) of 16S rRNA genes and transcripts between winter and summer (data not shown). The detected 8326 OTUs from the DNA samples represented a total of 45 phyla, while 11,105 OTUs from 49 phyla were found for the cDNA samples (see Supplementary Material). Archaea represented less than 0.04% of the total number of sequences at any sampling day at DNA and RNA level. The dominant phyla (>5% of total) in the DNA samples were *Proteobacteria* (22 ± 5% of the obtained DNA sequences [mean ± standard deviation, *n* = 36]), *Actinobacteria* (22 ± 4%*), Verrucomicrobia* (20 ± 6%), *Acidobacteria* (17 ± 3%), and *Gemmatimonadetes* (7 ± 3%) (Figure 2A). Together, these five phyla represented at least 74% of the bacterial community at any sampling day during the study period. In June, the abundance of *Cyanobacteria* peaked at 10 ± 5% of the community. At the cDNA level, the dominant potentially active phyla were *Proteobacteria* (39 ± 8% of the cDNA sequences), *Acidobacteria* (16 ± 5%), *Actinobacteria* (15 ± 5%), *Verrucomicrobia* (9 ± 3%), and *Cyanobacteria* (7 ± 14%) (Figure 2B).

### Seasonal variation in bacterial taxa

While the relative abundance of most phyla was stable over the year, the relative abundance of *Cyanobacteria* showed a pronounced seasonal variation from 0.17 ± 0.07% in January to 23 ± 6% of the cDNA sequences in the June sample. The relative abundance of the candidate phylum AD3 was larger in July-September (4–6%) compared to the remaining part of the year (0–2%), and the relative abundance of *Alphaproteobacteria* increased gradually from 16% in June to 33% in October and then decreased to 23% in November. Concomitantly with the decline in relative abundance of *Alphaproteobacteria*, the relative abundance of *Acidobacteria* increased from 11% in October to 23% in November.

**Figure 3:**
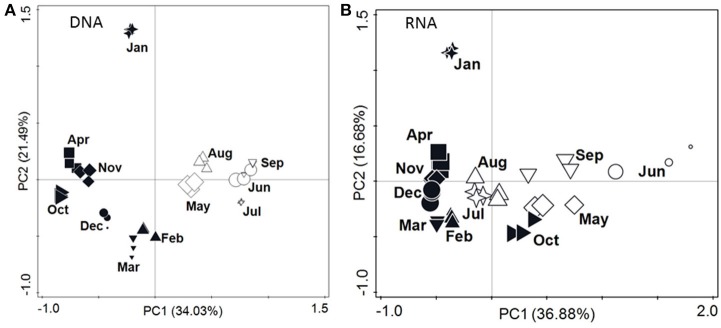
**Principal component plots of the relative abundance of OTUs inferred from the sequencing of 16S rRNA gene fragments at DNA level (A) and RNA level (B)**. The size of the symbols indicates the Shannon-Wiener index of the sample; the smallest symbols indicate an index of 3.65 and 2.99, while the largest symbols indicate an index of 4.30 and 4.67 for DNA level and RNA level, respectively. Closed symbols represent samples obtained when the soil was frozen (November through April). Note: Data points denoted “Apr” represent the soil core collected May 3rd 2010.

The first two principal components of the PCA analysis explained 56% of the variation within the OTU data from DNA and 54% of the variation from cDNA based data (Figure 3). At both the DNA and RNA level, the samples formed two major clusters; one consisting of samples from May through September, another of samples from October through April (Figure 3).

### Bacterial community structure and environmental parameters

In order to identify environmental parameters correlating with the bacterial community structure, redundancy analysis was performed (**Figure 5**) using soil physical and chemical parameters (Table 1) as the explanatory variables, and the bacterial diversity data at phylum and *Proteobacterial* class level (Figure 2) as the explained variables. At RNA level, the analysis revealed no significant effect of any of the measured soil parameters, while at DNA level 44.6% of the variation in bacterial community structure was explained by the analysis. pH and dissolved organic carbon correlated significantly (*P* < 0.05) with the data on bacterial community structure at DNA level explaining 24.6 and 20.0% of the variation, respectively. At DNA level, two of the numerically dominating bacterial groups, *Alphaproteobacteria* and *Acidobacteria*, correlated positively with dissolved organic carbon, while *Verrucomicrobia* correlated positively with pH (**Figure 5**). The analyses revealed no correlations between DNA-based community structure and soil depth or time of storage, but using time of storage as a covariate in the analysis increased *P* for the effect of pH from 0.031 to 0.063.

### Potential nitrogen-fixing and phototrophic bacteria

**Figure 4:**
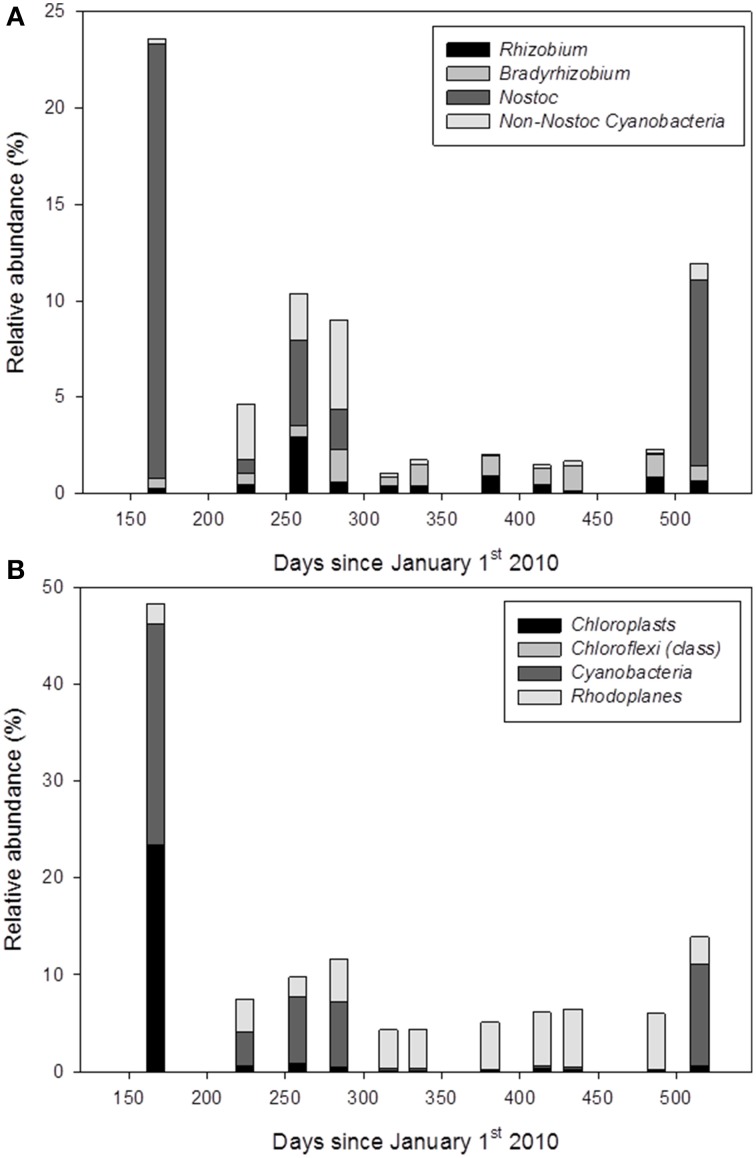
**Seasonal variation in the relative abundance of 16S rRNA transcripts of OTUs assigned to presumed nitrogen-fixing bacteria (A) and phototrophic bacteria and chloroplasts (B)**.

OTUs assigned to the following bacterial groups: *Rhizobium, Bradyrhizobium*, and *Cyanobacteria* were considered to be nitrogen-fixing bacteria. These groups consist exclusively of members with the ability to fix atmospheric nitrogen. For this analysis we excluded potential nitrogen-fixing OTUs from taxonomic groups, which encompass taxa not able to fix atmospheric nitrogen. Hence, we may underestimate the relative abundance of nitrogen-fixing bacteria. The relative abundance of nitrogen-fixing bacterial groups at RNA level peaked at 24% in June 2010 and decreased markedly from summer to early winter (Figure 4A), mainly due to a decrease in the relative abundance of *Cyanobacteria*. Potential phototrophic organisms were represented by OTUs assigned to *Cyanobacteria, Rhodoplanes, Chlorobi, Chloroflexi*, and chloroplasts (from algae and/or plants). The relative abundance of phototrophic organisms at RNA level ranged between 4.4% in November 2010 and 48% in June 2010 (Figure 4B).

The soil core collected on July 22nd 2010 did not contain the upper 1 cm of the soil profile. Since OTUs assigned to *Cyanobacteria* and other phototrophic organisms were assumed to originate mainly from the soil surface, data from this soil core were excluded from the estimation of the relative abundance of nitrogen-fixing and phototrophic bacteria. Accordingly, this soil core revealed a very low relative abundance of, e.g., *Cyanobacteria* (Figure 2).

## Discussion

### Bulk community structure

**Figure 5:**
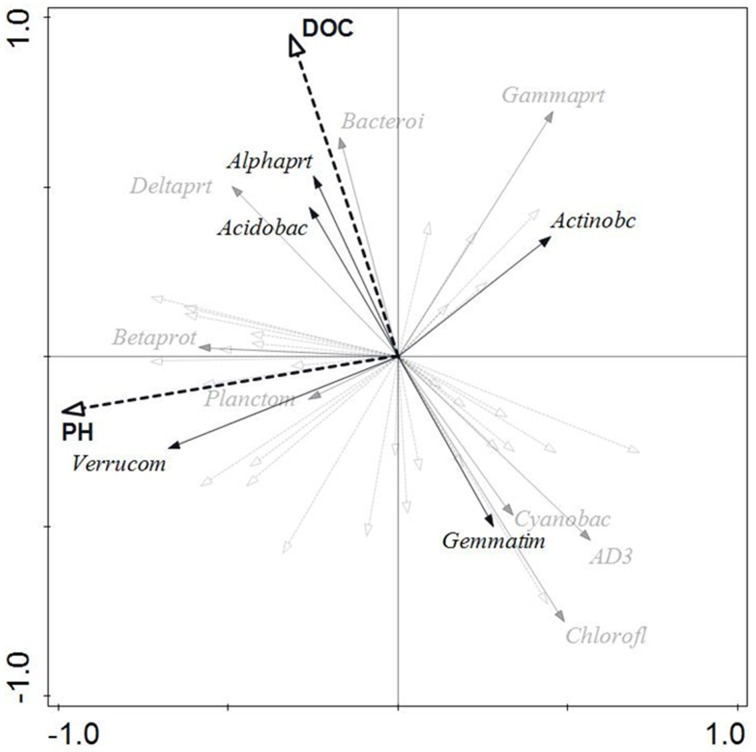
**Redundancy analysis biplot for bacterial phyla and proteobacterial classes at the DNA level in the 12 soil cores**. Dashed black arrows represent soil parameters with a significant (*P* < 0.05) influence on bacterial community structure. Solid black arrows represent dominant (>5% of the community) bacterial phyla and proteobacterial classes, while solid, gray arrows and hatched, gray arrows represent bacterial phyla and proteobacterial classes with a relative average abundance of 1–5% or <1%, respectively.

The dominance by *Proteobacteria, Actinobacteria, Verrucomicrobia, Acidobacteria*, and *Gemmatimonadetes* (Figure 2A) is in accordance with other studies of Arctic soils (Chu et al., 2010; Yergeau et al., 2010; Mackelprang et al., 2011; Tveit et al., 2013), except that the relative abundance of *Verrucomicrobia* in our samples (20 ± 6%) was about twice as large as in other Arctic soils. Also, the correlation between pH and the bacterial community structure (Figure 5) is in agreement with other studies from similar environments, including Arctic soil (Lauber et al., 2009; Gray et al., 2014; Kim et al., 2014; Siciliano et al., 2014). However, in contrast to a number of other studies we found no correlation between relative abundance of *Acidobacteria* and pH, and in contrast to Chu et al. (2010), we found that the relative abundance of *Actinobacteria* was negatively correlated with pH. This may be due to the narrow range of pH (4.7–5.6) covered by our study. The variations in soil pH showed no seasonal pattern (Table 1) and may partly be caused by spatial variation.

Many *Acidobacteria* are linked to soils with limited nitrogen availability (Fierer et al., 2007; Jones et al., 2009; Ramirez et al., 2010, 2012) and have been reported to respond negatively to nitrogen additions in Arctic soils (Campbell et al., 2010; Koyama et al., 2014). The *Acidobacteria* at our study site may be favored by limited nitrogen availability, which is indicated by a high relative abundance of potentially active nitrogen-fixing bacteria (Figure 4A; and Section Potential Nitrogen-fixing Bacteria).

### Potentially active community structure

At the RNA level, assumed to be representative of the potentially active component of the bacterial community, the same phylogenetic groups were dominant as for the DNA level except for a lower relative abundance of *Gemmatimonades* and a higher relative abundance of *Cyanobacteria*. These findings are comparable to other studies of Arctic soils (Männistö et al., 2009, 2013; Tveit et al., 2013; Tuorto et al., 2014). However, the ratio of 16S rRNA transcripts to gene copy numbers differed widely among the different phyla and *Proteobacterial* classes (Table 2). Even though the relationship between bacterial growth rates and copies of 16S rRNA transcipts differ among bacterial groups (Blazewicz et al., 2013) and different bacterial taxa may have different turnover of their 16S rRNA transcripts, the differences observed in this study may reflect different activity levels among bacterial groups with cells of *Deltaproteobacteria, Firmicutes*, and *Cyanobacteria* potentially being 100-fold more active than *Gemmatimonadetes*. Hultman et al. (2015) also observed a low ratio of relative abundance of *Gemmatimonadetes* in Alaskan active layer soil metatranscriptome relative to the metagenome.

The main potentially active bacterial groups at the study site may have different ecological roles (also see Sections Potential Nitrogen-fixing Bacteria and Potential Phototrophic Organisms). Several members of *Acidobacteria* are known to degrade plant- and microorganism-derived polysaccharides (Ward et al., 2009; Pankratov and Dedysh, 2010; Eichorst et al., 2011; Rawat et al., 2012), but we were not able to assign a specific ecological role to any of our acidobacterial OTUs. However, *Acidobacteria* and *Actinobacteria* are considered oligotrophs compared to *Proteobacteria*, which are versatile organisms and are considered copiotrophs (Fierer et al., 2003, 2007). This may explain the gradual increase in relative abundance of potentially active *Alphaproteobacteria* from June to October followed by a decrease from October to November. This shift between *Alphaproteobacteria* and *Acidobacteria* coincided with the onset of soil freezing. Even though freezing of soil water results in higher concentrations of dissolved organic carbon and nutrients as these are excluded during ice formation, freezing likely leads to an overall decrease in the availability of organic carbon and nutrients to bacteria due to a decrease in the volume of liquid water and lower diffusion rates.

### Seasonal variation of bacteria

The number of 16S rRNA gene copies was comparable to numbers reported by other studies on Arctic topsoil (e.g., Yergeau et al., 2010; Gittel et al., 2014). The copy numbers didn't show a seasonal pattern, which has also been shown for soils at lower latitudes (e.g., e Silva et al., 2012) and agrees with Wallenstein et al. (2007) suggesting that the abundance of bacteria in tundra soils is relatively stable throughout the year. In contrast, our principal component analysis of the OTUs revealed changes in community structure coinciding with thaw (May) and freezing (October), which has also been reported by Buckeridge et al. (2013), and a separate grouping of bacterial community structure from winter and summer samples (Figure 3A), which were obtained under widely different soil temperatures (Table 1). Temperature has been shown to affect microbial communities in temperate soils (e.g., Pettersson and Bååth, 2003; Castro et al., 2010). However, temperature and freeze–thaw cycles *per se* seem to have limited influence on the bacterial community structure of tundra soil and, e.g., *Acidobacteria* are resilient to freeze–thaw cycles (Männistö et al., 2009). Thus, seasonal changes in Arctic soil bacterial community structure may instead be linked to photosynthetically active radiation (PAR) and the availability of carbon substrates (Figure 5; and Rasche et al., 2010) that may undergo large seasonal shifts (Buckeridge and Grogan, 2008).

### Seasonal variation of the potentially active bacteria

As for DNA-based OTUs, the principal component analysis of the RNA-based OTUs showed a grouping of winter and summer samples (Figure 3B). McMahon et al. (2011) also detected seasonal shifts within the active bacterial communities of Arctic soils. In contrast, the copy number of 16S rRNA transcripts (Figure 1) didn't reveal a distinct seasonal pattern indicating potential bacterial activity during winter despite low soil temperatures, limited access to liquid water, and high osmolarity of liquid water. Different bacterial groups in permafrost soil have been shown to be active at different subzero temperatures down to −20°C (Tuorto et al., 2014), but in our study soil temperature *per se* didn't significantly affect the community structure of potentially active bacteria. Thus, osmolarity and liquid water limitations rather than low temperature *per se* may present greater stresses to the bacteria (Öquist et al., 2009; Ewert and Deming, 2014). Low temperature may limit, but not exclude bacterial activity, and microbial activity has been detected in soil samples at −18°C (Elberling and Brandt, 2003), *Actinobacteria* are able to sustain activity at low temperatures (Johnson et al., 2007), and specific species of bacteria are known to be active at very low temperatures (e.g., Bakermans and Nealson, 2004; Ayala-del-Río et al., 2010; Mykytczuk et al., 2013; Ewert and Deming, 2014). Also, in the Arctic, microbial activity during winter may continue at low temperatures and may contribute to degradation of organic matter (Welker et al., 2000). Since bacteria and archaea apparently are the main organisms responsible for organic matter turnover and greenhouse gas production in Arctic soils (Tveit et al., 2013), bacterial activity at low temperatures may have implications for our understanding of biogeochemical cycling of carbon and nitrogen in Arctic soils. Our data indicate that we need to address bacterial activity not only during the Arctic winter and shoulder seasons, but also as temperatures of permafrost soils increase (even when this doesn't lead to thawing of the soils).

### Potential nitrogen-fixing bacteria

We found a high relative abundance of 16S rRNA transcripts assigned to free-living (*Cyanobacteria)* and plant-associated nitrogen-fixing bacteria (*Rhizobium* and *Bradyrhizobium*) in or on the soil (Figure 4A). It should be noted that 16S rRNA transcripts are a proxy for potential bacterial activity but don't reveal the nature of the bacterial activity, i.e., if the activity was related to survival (e.g., preparation for dormancy or cold shock response, see Blazewicz et al., 2013, for a discussion) or was related to, e.g., nitrogen-fixing activity. Unfortunately, measurements of nitrogen-fixing activity weren't possible during the long winter period. However, most Arctic terrestrial ecosystems are characterized by nitrogen limitation (Tamm, 1991; Elser et al., 2007) and generally receive low amounts of atmospheric nitrogen deposition (<2 kg N ha^−1^ y^−1^) (Dentener et al., 2006). Thus, the high relative abundance of 16S rRNA transcript assigned to nitrogen-fixing bacteria indicates that the activity of nitrogen-fixing bacteria may be of ecological importance at the study site.

### Potential phototrophic organisms

16S rRNA gene- and transcript-based OTUs assigned to phototrophic organisms (mainly *Cyanobacteria*, and *Rhodoplanes*, and minor contributions from chloroplasts [presumed to originate from algae and plants], *Chlorobi* and *Chloroflexi*) showed a strong seasonal variation. The increase in abundance of chloroplasts and *Cyanobacteria* during early summer indicated growth of algae and *Cyanobacteria* on the soil surface during spring and early summer when snow had melted and incoming PAR was peaking. The low relative abundance of chloroplasts and *Cyanobacteria* in late summer and autumn was likely caused by drying of the soil surface during mid-summer, a decrease in PAR in autumn, and/or increased predation of the numerically dominant *Cyanobacteria* on the soil surface (Rønn et al., 2012). We have no estimates of the biomass of phototrophic bacteria, but the decline of *Cyanobacteria* and chloroplasts during summer may result in an important input of easily degradable organic matter to other soil microorganisms. A sudden input of easily degradable organic carbon has been shown to change the bacterial community structure in soils (Fierer et al., 2007) and to stimulate decomposition rates of old organic matter in permafrost subsoil (Wild et al., 2014).

Most of the gene fragments and transcripts assigned to phototrophic organisms in winter were assigned to *Rhodoplanes*. All described species of *Rhodoplanes* are known to be not only phototrophic, but also chemoorganoheterotrophic (Hiraishi and Ueda, 1994; Lakshmi et al., 2009; Okamura et al., 2009; Chakravarthy et al., 2012; Srinivas et al., 2014). The soil core collected July 22nd didn't contain the upper 0-1 cm of the soil profile. This soil core showed a similar relative abundance of 16S rRNA transcripts assigned to *Rhodoplanes* (4.3 ± 0.04%; average ± standard error of the mean) compared to the other eleven soil cores (4.1 ± 0.4%). This indicates that the *Rhodoplanes* at the study site are not phototrophic but are mainly chemoorganotrophic and may explain why these bacteria were active during the dark part of the year.

### Technical considerations

We are not aware of studies quantifying the turnover of 16 rRNA transcripts at temperatures relevant to this study, but we assume that the turnover rate decreases with decreasing temperature. Thus, 16S rRNA transcripts produced in winter may persist for longer time than 16S rRNA transcripts produced during summer. However, we were not able to estimate the fraction of the 16S rRNA transcript pool at a specific sampling date, which had been produced recently and thus reflected recent bacterial activity. The rapid decline in *Cyanobacterial* 16S rRNA transcripts during summer indicated a rapid turnover of 16S rRNA transcripts, but *Cyanobacteria* living on the soil surface may be prone to a rapid turnover due to, e.g., grazing by protozoa compared to bacterial phyla inhabiting the bulk soil. Also, we are not aware of studies investigating turnover of 16S rRNA transcripts bound to soil organic matter or minerals, which are known to preserve extracellular DNA in soils (Lombard et al., 2011), although it has been found that adsorption on clay minerals may extend the lifetime of rRNA exposed to RNases (Franchi and Gallori, 2005).

Each of our sampling dates is represented by only a single soil core. It may be argued that the comparison of non-replicated soil cores results in a study of spatial and not seasonal variation at the study site. This argument gains support from the fluctuations (between 2.4 and 8.7%; Table 1) in soil carbon content, which is presumed to be constant over the time span of 12 months. However, the grouping of the bacterial community structure at both DNA and RNA level into a summer and a winter cluster (Figure 3) indicates that the samples represent seasonal variations at the study site. Thus, we believe that our samples are representative of 1 year in the active layer of Arctic permafrost.

## Conclusion

The structure of both the bulk and the potentially active bacterial community in Svalbard active layer showed seasonal variation between summer and winter, and apparently sustained activity during winter. Phototrophic organisms on the soil surface and nitrogen-fixing bacteria may be responsible for important input of carbon and nitrogen to the soil.

### Conflict of interest statement

The authors declare that the research was conducted in the absence of any commercial or financial relationships that could be construed as a potential conflict of interest.

## Footnotes

^1^ Norwegian Meteorological Institute. (2014). Data retrieved from http://eklima.met.no/; Station 99840, Svalbard Lufthavn.
